# Psychometric Properties of the German Translated Version and Adaptation of the Food Craving Inventory

**DOI:** 10.3389/fpsyg.2017.00736

**Published:** 2017-05-10

**Authors:** Ernesto Tarragon, Jakob Stein, Jobst Meyer

**Affiliations:** Department of Neurobehavioral Genetics, Institute of Psychobiology, University of TrierJohanniterufer, Germany

**Keywords:** eating behavior, obesity, psychometric validation, factor analysis

## Abstract

Dysfunctional eating behavior is a major risk factor for developing all sorts of eating disorders. Food craving is a concept that may help to understand better why and how these and other eating disorders become chronic conditions through non homeastatically-driven mechanisms. As obesity affects people worldwide, cultural differences must be acknowledged to apply proper therapeutic strategies. In this work, we adapted the Food Craving Inventory (FCI) to the German population. We performed a factor analysis of an adaptation of the original FCI in a sample of 326 men and women. We could replicate the factor structure of the FCI on a German population. The factor extraction procedure produced a factor solution that reproduces the four factors described in the original inventory, the FCI. Our instrument presents high internal consistency, as well as a significant correlation with measures of convergent and discriminant validity. The FCI-Deutsch (FCI-DE) is a valid instrument to assess craving for particular foods in Germany, and it could, therefore, prove useful in the clinical and research practice in the field of obesity and eating behaviors.

## Introduction

For a time now, obesity has been a remarkably significant health issue worldwide. What initially was thought a simple problem of calories in vs. calories out has turned into a complex, multifactorial condition influenced by genes as well as social environment (Locke et al., 2015; Díez et al., 2016; Perry et al., 2016). Moreover, some researchers have demonstrated that the brain regions that are more strongly activated in drug abusers when drug cues are presented, are similarly activated in obese people when food cues are presented (Pelchat et al., 2004; Tang et al., 2012). Given that the behavioral response to reinforcers (whether natural or artificial) is governed by the same neuronal pathways, this similarity between the brain activation patterns mentioned above gave support to the hypothesis of food addiction. However, despite this term being broadly adopted by media and the general population, this concept is still controversial in scientific terms and yet subject to debate.

In line with the evidence that supports food addiction as a valid phenotype and phenomenon (Davis et al., 2011), experiencing craving represents a major feature (or symptom) of this construct. The attribution of such a pivotal role to craving by most of the addiction research suggests that this attribute is real rather than a mere theoretical construct (Meule and Kübler, 2012; Potenza and Grilo, 2014; Chao et al., 2016). And as it happens in drug addiction, (food) cravings are frequently reported among individuals that fit the food addiction descriptors, as defined by the DSM and other instruments specifically developed to explore this condition (Pursey et al., 2014), like the Yale Food Addiction Scale (YFAS) (Gearhardt et al., 2009; Meule and Gearhardt, 2014). The drug literature commonly differentiates between subjective and behavioral aspects of craving. Although when it comes to food, this dichotomy seems problematic, pragmatically speaking, as a behavioral characterization of craving would be indistinguishable from the behavioral characterization of physiological hunger (Hill, 2007). Therefore, it is common to use the term as a synonym of the subjective experience only, understood as “an urgent desire, longing, or yearning for a particular substance” (Hormes and Rozin, 2010).

The experience of food craving has been associated with obesity (Potenza and Grilo, 2014) and overeating (Scherwitz and Kesten, 2005). Interestingly, it seems to be common across cultures as well, and various methods have been developed to evaluate it in the past years (Cepeda-Benito et al., 2000; Meule et al., 2014). The Food Craving Inventory (FCI) (White et al., 2002) seems particularly interesting, given that differently from other instruments, it focuses on particular foods. This inventory divides its 28 items into four subscales (“Sweets,” “High fats,” “Carbohydrates/starches,” and “Fast food fats”) and has proved valid in evaluating food craving. After the validation of the original instrument, other studies were able to replicate these results as regards both structure and category. The adaptation to the UK population by Nicholls and collaborators (Nicholls and Hulbert-Williams, 2014) displayed the same four factors as the original FCI. The Spanish (Lobera et al., 2010) and Japanese (Komatsu, 2008) versions present some dissimilarities, with three- and five-factor solutions, respectively. However, they all show in general a clear discrimination in craving for different food categories, with “Sweets,” “High fats,” and “Fast food” consistent across the three studies.

In agreement with this, the aim of this work was to come up with a psychometrically solid version of the FCI adapted to the German population, able to replicate the factor structure and food categories in the original and later versions. Like many other countries, Germany presents concerning rates of overweight and obesity, with more than half of the population overweight, and almost one in every four adults being obese (Schienkiewitz et al., 2012). Identifying the same type of craving in another population, with noticeable cultural differences as regarding eating habits, would add support to the idea of food craving as a true phenomenon and might help to understand it better and narrow its definition. In addition, sex differences in craving to different foods have been reported (Lobera et al., 2010). Research shows that these differences can occur not only with respect to food items, but also in intensity (Hallam et al., 2016). Given that these differences could also have an impact on dietary interventions, we additionally made sex comparisons in the craving score.

## Methods

### Participants

Sixty participants (21 males and 39 females, from age 18 to 41) were recruited from the University of Trier via e-mail digest to fill the preliminary version of the FCI-Deutsch (FCI-DE) in a pilot study. A document explaining the study was handed to the volunteers, which they signed when they agreed to the terms of the procedure. Another sample of 326 participants was used to perform the exploratory factor analysis (EFA) and confirmatory factor analysis (CFA). The recruitment of the 126 men (35.64 ± 13.86, years old) and 201 women (31.54 ± 12.95, years old) was carried out also by e-mail digest, and advertisement in various social networks. Participants were excluded from the analysis when under dietary restriction, either for losing/gaining weight purposes, illness, or personal beliefs that significantly affect attitudes toward food (i.e., veganism). To this purpose, participants were asked directly if they practiced such restriction [“Do you practice any sort of diet that requires the absence of a particular food or food group, either by medical reasons or personal choice (i.e., lactose intolerance, veganism)? If yes, please, specify”]. All participants were informed of the nature of the study and agreed voluntarily to participate. All of the procedures were conducted following ethics procedures approved by the University of Trier Ethics Committee. This study raised no ethical concerns.

### Instruments

#### Development of the FCI-DE

The first version of the FCI included not only the subjective scale but also two behavioral measures (White et al., 2002), one that accounts for the frequency of giving in to the food craving and another one assessing the difficulty of resisting the feeling. In order to remain as close as possible to the original procedure, we included this second scale in the pilot study.

A straight English-to-German translation of the original 28 items was carried out initially. A closer look at the translated items made clear cultural discrepancies regarding various foods. Following discussion with naturals from the country led us to adapt some items into more regionally recognizable alternatives and to include some typically German foods, despite them lacking correspondence in the original inventory. Contributors to this discussion were two men and five women, between 22 and 60 years old, born and raised in Germany, which guaranteed a good knowledge of traditional and more actual dietary habits and foods in the country. As consequence of this discussion, the items “Fried chicken,” “Hot dog,” “Corn bread,” “Cinnamon rolls,” and “Grains” were substituted by “Frikadellen” (meatballs), “Döner” (as in Doner Kebab), “Marzipan,” “Süsse Backwaren” (sweet pastries), and “Haferflocken” (rolled oats). Also, we combined the “Biscuits” and “Cookies” items into a single option (“Keks”), and differentiate “Pancakes, waffles” into “Pfannkuchen” and “Waffeln.” In addition, we included the items “Brezel” (pretzel), “Belegtes Brötchen” (a sort of sandwich with various fillings and types of bread), “Crackers,” “Gesalzene Erdnüsse” (salty peanuts), “Honig” (honey), “Müsli” (muesli), and “Nussnougatcreme” (chocolate hazelnut spread). The item “Gravy” was eliminated, as we did not find an adequate alternative to it. This preliminary version consisted of two identical scales (subjective craving and giving in) of 34 items in total, ranging from 1 (“Never”) to 5 (“Always/Almost always”), and included as well a question about dietary restraint, currently and/or during the last month (Annexe 1 in Supplementary Material).

#### Convergent validity

Following the procedure from the original FCI validation study, we chose the Three Eating Factor Questionnaire (Stunkard and Messick, 1985) as a measure of convergent validity. We used the German version, the Fragebogen zum Essverhalten (FEV), adapted by Pudel and Westenhöfer (1989). As in the original work by White et al. (2002), the Disinhibition and Hunger scales were used to assess convergent validity, and the Cognitive control (restraint) scale was used as a discriminant validity measure.

### Procedure

Participants in the pilot study were invited to come to the laboratory facilities to take part in another, related study. After a brief introduction and signing the informed consent, the preliminary version of the FCI-DE and the FEV were handed to each participant. The total time to complete both questionnaires was 10–15 min. One participant had to be excluded from the analysis due to his refusal to complete one of the questionnaires. Participants were rewarded for their time with 5 euros or 15-min credit value stickers. Only the subjective scale was later used for the validation study. For this, both the corrected version of the FCI-DE and the FEV were digitalized and uploaded as online questionnaires. Participants could access both surveys through a link at the bottom of a brief text that included a short description of the project and instructions on how to proceed. Specifics on anonymity and data treatment were also detailed. Answers were automatically registered and saved into a spreadsheet, which allowed further import into the analysis software.

### Data analysis

An exclusion criterion was established, and foods were eliminated from the scale if marked as “never” or “rarely” craved more than 85% of the occasions. Given the non-continuous nature of the items, internal consistency was evaluated by calculating composite reliability (CR) for congeneric measures (Raykov, 1997). Pearson correlation coefficient was used to explore associations between items. Principal Axis Factoring and Promax rotation was performed to examine the factorial structure of the questionnaire. Consistent with the original inventory, we set a coefficient cut-off point of 0.45. Items below this value after the extraction, or showing an inter-item correlation below 0.2 were eliminated from the analysis. Factor retention was determined by parallel analysis and Eigenvalue significance, using the 95 percentile as the cut-off point (O'Connor, 2000). The EFA and the group comparison analysis were conducted using the Statistical Software Package SPSS (version 23; IBM SPSS, Chicago, IL). The confirmatory factor analysis was carried out using the EQS 6.3 software for structural equation modeling (Multivariate Software, Inc., California, USA).

## Results

According to our data, 25% of our sample (*n* = 79) was overweight (BMI > 25), which is consistent with the data available on overweight and obesity rates in Germany (Schienkiewitz et al., 2012). From these 25%, women represented 62% (*n* = 49) and men 38% (*n* = 30). Sample characteristics are shown in Table 1.

**Table 1:** **Sample characteristics**.

	***N***	**Age (mean ± sd)**	**High (cm) (mean ± sd)**	**Weight (Kg) (mean ± sd)**	**BMI (mean ± sd)**
Men	126	35.64 ± 13.86	181.26 ± 10.38	76.66 ± 15.93	23.01 ± 6.21
Women	201	31.54 ± 12.95	168.11± 8.37	73.01± 16.92	25.83 ± 6.06

The analysis of the results of the pilot study showed that there were no differences between subjective craving and giving in scales for any of the items. Therefore, following the procedure from the original FCI, we carried on the analysis with the subjective craving scale alone.

The factor structure of the FCI-DE was explored and considered using various criteria. Bartlett's test of sphericity (χ^2^ = 2563.85, *p* < 0.001) and the Kaiser-Meyer-Olkin index (KMO = 0.861) supported pursuing the analysis of underlying factors. Standardized root mean square residual was below 0.08, standard value for fitting-model acceptance. Additionally, the diagonals of the anti-image correlation matrix and Communality values were all over 0.75 and 0.3, respectively. Six items, “Fried fish,” “Bacon,” “Crackers,” “Marzipan,” “Salty nuts,” and “Rolled oats” were excluded from the final list of items following the exclusion criteria. Internal consistency analysis of the 28-item scale showed a CR of 0.887. All this confirmed the adequacy of the 28 items for further factor examination.

The number of factors to retain was determined by parallel analysis (Lautenschlager, 1989) in 163 randomly selected cases (half of the sample; 55 men and 108 women). Seven factors showed Eigenvalues greater than 1, although only four were statistically significant. These four factors explained 50.47% of the total variance, and contained 11 (21.86%), 8 (12.99%), 5 (8.68%), and 4 (6.92%) items (Table 2). Foods loading on the different factors shared nutritional properties that led us to label the categories as “Sweets” (CR = 0.867), “Starches” (CR = 0.821), “High fats” (CR = 0.891), and “Fatty/Salty carbohydrates” (CR = 0.742).

**Table 2:** **Structure matrix of the FCI-DE**.

**Item**	**Factor**			
	**1**	**2**	**3**	**4**
Cake	**0.720** (0.712)			
Pastries	**0.713** (0.731)			
Brownie	**0.697** (0.607)			
Chocolate	**0.680** (0.749)			
Biscuits	**0.653** (0.666)			
Chocolate hazelnut spread	**0.620** (0.637)			
Waffle	**0.569** (0.531)			
Sweets	**0.557** (0.745)			
Ice cream	**0.509** (0.539)			
Pancakes	**0.490**			
Donuts	**0.481**			
Rolls		**0.715** (0.724)		
Rice		**0.676** (0.721)		
Pasta		**0.646** (0.639)		
Potatoes		**0.635** (0.611)		
Sandwich		**0.619** (0.665)		
Muesli		**0.548** (0.579)		
Honey		**0.497** (0.548)		
Pretzel		**0.483**		
Meatballs			**0.782** (0.691)	
Sausages			**0.764** (0.774)	
Steak			**0.733** (0.720)	
Hamburger			**0.690**	(0.580)
Doner kebab			**0.638**	(0.501)
Pizza				**0.769** (0.788)
Chips				**0.699** (0.733)
Crisps				**0.646** (0.723)
White bread				**0.456**

*Factor 1, Sweets; 2, Carbohydrates/starches; 3, High fats; 4, Fast food fats. Bold numbers represent the loadings in the EFA. Numbers in brackets represent item loadings of the CFA after re-examination*.

A CFA using a structural equation modeling procedure was performed afterwards in the other half of the sample (71 men, 94 women). The analysis confirmed the four-factor structure obtained in the EFA. Goodness of fit was estimated by Maximum Likelihood method. Both sphericity (χ^2^ = 2475.47, *df* = 378, *p* < 0.001) and sampling adequacy criteria were met. Other fit indices, like Bentler-Bonnet non-normed index (=0.723) and the Comparative Fit Index (CFI = 0.802) suggested that the model could be improved (Hu and Bentler, 1999). A more thoughtful examination of the individual items was carried out. We found that four items, “pretzel,” “pancakes,” “donuts,” and “white bread” loaded into more than one category, and were thus subtracted from the inventory. After removing these items a CFA was again conducted and fit indices improved (Bentler-Bonnet non-normed index = 0.872, RMSEA = 0.079, CFI = 0.911).

Four factors accounted for 52.89% of the total variance. Concretely, 24.30% of the total variance was explained by factor 1, 13.74% was explained by factor 2, and 7.91 and 6.95% of the total variance by factors 3 and 4, respectively. The removal from these items provoked a reconfiguration of the items loading in each factor. The “Sweets” and “Starches” factors remained unchanged, except for the items respectively eliminated from each one (“pretzel” and “pancakes” and “donuts”). The “High fats” counted now with only three items; “meatballs,” “steak,” and “sausages.” The “Fatty/Salty carbohydrates” factor was re-labeled as “Fast food fats,” given that together with “pizza,” “chips,” and “crisps,” two more items loaded within; “doner,” and “hamburger.” The CR analysis of the individual subscales indicated good internal consistency, with factors showing a CR of 0.890 (“Sweets”), 0.831 (“Starches/Carbohydrates”), 0.773 (“High fats”), and 0.802 (“Fast food fats”). Table 3 shows the correlations between the four subscales.

**Table 3:** **Reliability and correlation matrix of FCI-DE subscales**.

	**Sweets**	**Carbohydrates/Starches**	**High fats**	**Fast food fats**
Internal consistency (CR)	0.890	0.831	0.773	0.802
Sweets				
Carbohydrates/Starches	0.209^**^			
High fats	0.100	0.315^**^		
Fast food fats	0.241^**^	0.323^**^	0.366^**^	

*CR, Composite Reliability*.

** *Significant at p < 0.01*.

Convergent validity was also evaluated. Table 4 shows the correlation values between the FCI-DE four factors and the three subscales of the FEV. Our results show a positive, significant correlation between “Disinhibition” and the “Sweets” (*r* = 0.141, *p* = 0.041) factor. “Hunger” correlates significantly with “Fast food fats” (*r* = 0.198, *p* = 0.007) factors. No correlation was found between the “Cognitive control” and any of the FCI-DE factors. Correlation between body mass index (BMI) and subscale scores of the inventory was explored as well, but no significant results were obtained.

**Table 4:** **Convergent validity correlations**.

		**FEV**		
	**BMI**	**Cognitive control**	**Disinhibition**	**Hunger**
Sweets	0.141	0.041	0.141^*^	0.082
Carbohydrates/Starches	−0.035	0.018	0.126	0.107
High fats	−0.048	0.026	0.044	0.104
Fast food fats	−0.088	0.103	0.092	0.198^**^

*Correlation between the FCI-DE subscales, BMI and the three factors from the FEV*.

* *Significant at p < 0.05*,

** *significant at p < 0.01*.

We also performed a non-parametric comparison with Bonferroni correction for multiple comparisons on sex, relating to items and subscales craving. Mann-Whitney U test for independent samples showed a significant difference in craving score in “potatoes” (1.66 ± 0.87 vs. 1.98 ± 0.138, *p* = 0.025, *d* = 0.350) and “steak” (1.52 ± 0.84 vs. 1.88 ± 0.15, *p* = 0.041, *d* = 0.314). We did not find differences between overweight and normal weight people with respect subscale scores.

## Discussion

In this work, we attempted to develop a valid instrument to identify craving for particular foods in the German population. To this purpose, we translated the FCI (White et al., 2002) to the cultural idiosyncrasy of Germany, adding and modifying items as necessary to better fit the target population. We kept 27 items from the original FCI, from which seven were substituted by a more culturally appropriate option. In the first revision of the FCI-DE, we showed that the subjective and behavioral scale presented no differences as regards item scorings. This is coherent with what White and collaborators found when analyzing the first version of the inventory. Thus, following their procedure, we disregarded the behavioral scale as well. Additionally, three foods that corresponded to adapted items were removed, leaving 24 of the 28 items from the original instrument. After refinement, the final form of the FCI-DE counted with 24 items.

The preliminary factor structure of the corrected version of the FCI-DE was similar to that found in in the original FCI and other adaptations (White et al., 2002; Nicholls and Hulbert-Williams, 2014). The confirmatory analysis supported this four-factor arrangement, although one of the categories was relabeled to better describe the items within (Fatty/Salty carbohydrates). In an attempt to improve the model, however, the reconfiguration of the factor structure led to item loadings remarkably similar to those from the American FCI. Hence, we maintained the original name of the factors; “Sweets,” “Carbohydrates/Starches,” “High fats,” and “Fast food fats.”

Comparing both instruments in a scale-by-scale level, several similarities appear. The four-factor solution is reproduced, with equivalent or same items loading onto each. The “Sweets” subscale of our instrument, for example, includes all the items present in the original “Sweets” subscale from the FCI, and the cultural alternatives to many foods load onto their hypothesized category (i.e., doner kebab, substituting hot dogs). The internal consistency and reliability analysis of the FCI-DE show that this instrument is psychometrically stable. The four subscales present good correlation levels with each other. Furthermore, our inventory correlates with the three factors of the FEV as expected. This suggests the existence of an underlying construct (food craving) with unique features and supports the FCI-DE as a valid instrument to assess craving frequency.

However, we must address limitations in the study. First and foremost, alas acceptable, the fit indices were not as good as desirable. One of the reasons could be the sample size. Our sample size of 326 participants (EFA + CFA) falls into what some authors consider appropriate with an inventory with such characteristics as this one (MacCallum et al., 1999). Also, it seems to be large enough to reproduce the results obtained by White et al., and other adaptations of the FCI. A larger sample may help to address the issue of goodness of fit. Another reason could be that some of the items are rarely eaten alone. For example, bread rolls, pasta, or muesli, are often mixed with sauces, fillings and toppings. The combination of different foods may affect their categorization, therefore influencing how well adapted they are to the model. In relation to this, we also have to mention that despite the popularity of the items in the inventory, no back-translation was used after translating the foods to German. Given that the people involved in this task were all part of the scientific community and displayed good knowledge in English, we believed that this second step was not mandatory.

Second, given the recruitment method there is some information missing that could help to interpret the results better. For instance, eating customs may differ significantly among regions across Germany, and we don't know the location of each participant. Also, the pilot study was conducted in the context of another experiment that forbid the participants to eat anything 3 hours before coming to the laboratory. Some participants reported a certain level of difficulty answering the inventory because “it is hard to think about it without having eaten anything in 3 h” (paraphrasing).

Previous research has proven a relation between food cravings and BMI (Franken and Muris, 2005), as well as differences in food cravings between healthy and obese people (Abilés et al., 2010; Meule and Kübler, 2012). We explored this relation as well, but since we found no differences between overweight and normal weight people as regards craving scores, we could not replicate these findings. However, since we relied on self-report information relating to height and weight and so the information on the BMI is calculated from these parameters, these values might not be entirely accurate. Thus, as with every study, results must be interpreted cautiously. Future replication should follow, and it would be desirable that other researchers try to replicate our findings using the scale we developed.

The majority of strategies fighting obesity are based on the nutritional aspects of the intervention. And despite the apparent value and necessity of nutritional education, psychological factors must not be neglected. Like others before us, we show that craving can occur not only toward “junk food” and can be subdivided into different food categories. This specificity implies that craving might not be a consequence but a sign of a potential dysfunctional relationship with food (Greeno et al., 2000; White and Grilo, 2005). We believe that targeting food cravings in particular, rather than food addiction, would be useful in elaborating individual dietary intervention plans. Indeed, there is evidence suggesting that craving acts as the main motivational factor for consolidating unhealthy habits (Tiffany and Wray, 2012), and it is pointed out also as the primary responsible for relapse in people attempting modifications in their dietary habits (Moreno et al., 2009). Therefore, as the potential risk factor that cravings represents, we trust that every tool available to better understand this construct should be welcome. With its psychometric validity proved here, we deem that the properties of the FCI-DE could be helpful in this regard.

## Author contributions

ET participated in the design, data collection, writing, and discussion of this manuscript. JS participated in the design, data collection of this manuscript. JM participated in the design, and discussion of this manuscript.

## Funding

ET was financially supported by the Alexander von Humboldt-Stiftung under the “Humboldt Research Fellowship for Postdoctoral Researchers” programme during the development of this work.

### Conflict of interest statement

The authors declare that the research was conducted in the absence of any commercial or financial relationships that could be construed as a potential conflict of interest.
