# Supplementary material for: Psychometric Properties of the German Translated Version and Adaptation of the Food Craving Inventory
Source: Front Psychol. 2017 May 10;8:736. doi: 10.3389/fpsyg.2017.00736 (PMC5423968; doi:10.3389/fpsyg.2017.00736)
Supplement: Supplementary file 1 [file DataSheet1.docx]

Annexe 1. Original 34-item FCI-DE inventory.

| „Craving“ ist ein Fachbegriff aus der Psychologie, der das nahezu unbezwingbare Verlangen einer Person umschreibt, ein bestimmtes Nahrungsmittel zu sich zu nehmen. Bitte markieren Sie, wie oft Sie im letzten Monat „Craving“ nach den folgenden Lebensmitteln gefühlt haben.  *(“Craving” is a concept from Psychology that refers to one’s unstoppable desire to consume a particular food. Please indicate how often you felt “craving” for the following foods in the last month.)* | | | | | |
| --- | --- | --- | --- | --- | --- |
|  | Nie  (*Never*) | Selten  (*Rarely*) | Manchmal  (*Sometimes*) | Häufig  (*Often*) | Immer / Fast immer  (*Always /Almost always*) |
| Frikadelle  (*Meatballs*) | □ | □ | □ | □ | □ |
| Wurst  (*Sausages*) | □ | □ | □ | □ | □ |
| Backfisch  (*Fried* *fish*) | □ | □ | □ | □ | □ |
| Frühstücksspeck  (*Bacon*) | □ | □ | □ | □ | □ |
| Marzipan | □ | □ | □ | □ | □ |
| Döner  (*Doner* *kebab*) | □ | □ | □ | □ | □ |
| Steak | □ | □ | □ | □ | □ |
| Brownie | □ | □ | □ | □ | □ |
| Keks  (*Biscuits*) | □ | □ | □ | □ | □ |
| Süßigkeiten  (*Sweets*) | □ | □ | □ | □ | □ |
| Schokolade  (*Chocolate*) | □ | □ | □ | □ | □ |
| Donuts | □ | □ | □ | □ | □ |
| Kuchen  (*Cake*) | □ | □ | □ | □ | □ |
| Süße Backwaren  (*Sweet* *pastries*) | □ | □ | □ | □ | □ |
| Eiscreme  (*Ice* *cream*) | □ | □ | □ | □ | □ |
| Brötchen  (*Rolls*) | □ | □ | □ | □ | □ |
| Pfannkuchen  (*Pancakes*) | □ | □ | □ | □ | □ |
| Waffel  (*Waffles*) | □ | □ | □ | □ | □ |
| Toastbrot  (*Toast* *bread*) | □ | □ | □ | □ | □ |
| Reis  (*Rice*) | □ | □ | □ | □ | □ |
| Ofenkartoffel  (*Potatoes*) | □ | □ | □ | □ | □ |
| Nudeln  (*Pasta*) | □ | □ | □ | □ | □ |
| Haferflocken  (*Rolled* *oats*) | □ | □ | □ | □ | □ |
| Hamburger | □ | □ | □ | □ | □ |
| Pommes frites  (*Chips*) | □ | □ | □ | □ | □ |
| Chips  (*Crisps*) | □ | □ | □ | □ | □ |
| Pizza | □ | □ | □ | □ | □ |
| Brezel  (*Pretzel*) | □ | □ | □ | □ | □ |
| Belegtes Brötchen  (*Sandwiches*) | □ | □ | □ | □ | □ |
| Cracker | □ | □ | □ | □ | □ |
| Gesalzene Erdnüsse  (*Salty* *nuts*) | □ | □ | □ | □ | □ |
| Honig  (*Honey*) | □ | □ | □ | □ | □ |
| Müsli  (*Muesli*) | □ | □ | □ | □ | □ |
| Nussnougatcreme  (*Chocolate* *hazelnut* *spread*) | □ | □ | □ | □ | □ |
